# Sulfhemoglobinemia in a 53-Year-Old With a History of Phenazopyridine Misuse

**DOI:** 10.7759/cureus.40944

**Published:** 2023-06-25

**Authors:** Matthew Soderstrom, Daniel V Foster, Zachariah S Hamidi, Jess T Anderson

**Affiliations:** 1 Department of Internal Medicine, Brooke Army Medical Center, San Antonio, USA; 2 Department of Pulmonary and Critical Care Medicine, Brooke Army Medical Center, San Antonio, USA

**Keywords:** toxicology, adverse reaction, dyshemoglobinemia, sulfhemoglobinemia, phenazopyridine

## Abstract

Sulfhemoglobin is formed by the irreversible bonding of sulfur atoms to the heme molecule. Oxygen is then unable to bind the heme molecule, rendering the hemoglobin molecule unable to carry oxygen. The most common etiology of sulfhemoglobinemia is the use/misuse of sulfur-containing medications such as AZO. Unlike methemoglobin, sulfhemoglobin, due to its irreversible binding, has no antidote, and the treatment is ultimately supportive. We present a case of a 53-year-old female who presented to the emergency room endorsing dysuria and was noted to have abnormally low oxygen saturation (SpO2) despite having high arterial oxygen pressure (PaO2) on blood gas. History was significant for dysuria developed while traveling and the use of over-the-counter AZO four times daily for the past 10 days. She was diagnosed with a presumed dyshemoglobinemia and, upon return of send-out labs, was confirmed to have sulfhemoglobinemia attributed to phenazopyridine. This case highlights the importance of the recognition of potential dyshemoglobinemias and consideration of sulfhemoglobinemia as a potential causative etiology, especially in patients taking sulfur-containing medications.

## Introduction

Cyanosis refractory to the administration of supplemental oxygen is the expected presentation of a patient with dyshemoglobinemia. Methemoglobinemia and sulfhemoglobinemia - the two most common dyshemoglobinemias - share many clinical features and yet have fundamentally different pathophysiologic mechanisms and management principles. Physicians must rely on the clinical history with close attention paid to the use of non-prescription medications when evaluating and treating a patient with presumed acquired dyshemoglobinemia. Sulfhemoglobinemia, in contrast to methemoglobinemia, causes a rightward shift in the oxygen-hemoglobin dissociation curve (as opposed to a left shift) and cannot be identified on routine blood gas analysis. Further, there is no medical antidote for the treatment of sulfhemoglobinemia, whereas most hospitals can readily access methylene blue for the reversal of clinically significant methemoglobinemia [1]. Here, we discuss a case of sulfhemoglobinemia in a patient that presented to our emergency department (ED) after the recent initiation of over-the-counter phenazopyridine for the treatment of dysuria.

This article was previously presented as a case abstract at the 2022 CHEST Scientific Meeting on October 17, 2022.

## Case presentation

A 53-year-old woman with a history of recurrent urinary tract infections (UTI) presented to the ED with the chief complaint of dysuria and intermittent nausea for the past seven days. She was unable to seek medical evaluation or care, as she had been traveling at the time of symptom onset. She reported using over-the-counter phenazopyridine (brand name AZO) for treatment, with an initial improvement in her symptoms. However, the patient soon began to experience worsening dysuria and increased her use of phenazopyridine to four times daily for four days prior to presenting. At the time of presentation, the review of systems otherwise included no reported history of fever, flank pain, chest pain, dyspnea, peripheral edema, palpitations, or orthopnea. The patient reported taking no prescription medications and denied the use of any other over-the-counter drugs or supplements.

In the ED, the patient was in no acute distress, with initial vital signs significant only for a pulse oximetry reading of 88%. Physical exam was notable for the sharply demarcated distal pallor of the bilateral fingers suggestive of Raynaud’s phenomenon. The patient denied any pain or paresthesia in her fingers and explained that she had never had these symptoms previously. Due to initial concern that poor distal perfusion of the fingers was interfering with the pulse oximeter, the device was then placed on the patient’s ear without improvement in the oxygen saturation (SpO2). She was then treated with progressively increasing amounts of supplemental oxygen until she began to show pulse oximetry readings of 92% at 30 liters and 100% fraction of inspired oxygen (FiO2) via a high-flow nasal cannula.

The patient did not endorse any respiratory distress throughout the initial evaluation. The pulmonary exam was without positive findings. Initial plain film chest imaging was similarly unrevealing, and a 12-lead electrocardiogram (ECG) demonstrated normal sinus rhythm at a rate of 90. Complete blood count was notable for mild leukocytosis and stable mild microcytic anemia. Both troponin-T and pro-brain natriuretic peptide levels were within normal limits. The coagulation panel and D-dimer were both within normal limits, and the hepatic function panel revealed mildly elevated aspartate aminotransferase and alanine aminotransferase at 50 U/L and 42 U/L, respectively. See Table 1 for the key laboratory values on presentation. Arterial blood gas (ABG) was performed after she had been placed on a high-flow nasal cannula and had a slight respiratory alkalosis with normal lactate. At this time, the medical intensive care unit (MICU) was consulted for admission.

**Table 1:** Key Laboratory Values on Presentation L – liter, g – grams, dL – deciliter, ng – nanograms, mL – milliliter, pg – picograms, U – units, mmHg – milliliters of mercury, mmol – millimole

Lab Name	Lab Value	Reference Range
White Blood Count	12.5X10^9^/L	4.5-10X10^9^/L
Hemoglobin	11.0 g/dL	12.1-15.1 g/dL
Troponin-T	<0.01 ng/mL	<0.01-0.03 ng/mL
Pro-brain natriuretic peptide	39 pg/mL	<400 pg/mL
Aspartate aminotransferase	50 U/L	0-35 U/L
Alanine aminotransferase	42 U/L	4-36 U/L
pH	7.47	7.35-7.45
Partial pressure of oxygen	241 mmHg	75-100 mmHg
Partial pressure of carbon dioxide	35 mmHg	35-45 mmHg
Lactate	0.91 mmol/L	0.5-1.6 mmol/L

Due to the fact that the patient was treated with supplemental oxygen for several hours prior to obtaining an ABG, the care team was unable to confirm true hypoxia prior to the patient’s admission to the MICU. However, given her persistently low SpO2 readings despite high levels of supplemental oxygen, the MICU team considered dyshemoglobinemia high on the differential diagnosis. Unfortunately, the hospital lab was unable to process the necessary studies to confirm either methemoglobinemia or sulfhemoglobinemia. The patient’s blood samples were sent to outside laboratories for further analysis. Further etiologies for her hypoxia, including pulmonary shunting and arteriovenous malformations, were evaluated with transthoracic echocardiogram and CT angiography. These studies were both grossly normal. Within 48 hours of being hospitalized, the patient was weaned off of supplemental oxygen and soon had pulse oximetry readings greater than 90% on room air. Throughout her stay, she remained asymptomatic. Initially, the care team believed the use of over-the-counter phenazopyridine for seven days (exceeding the maximum treatment duration of two days) with increased doses taken in the last four days prior to presentation was the most likely etiology of her suspected dyshemoglobinemia. She was empirically treated for methemoglobinemia with methylene blue, and her urinary tract infection was treated with a one-time dose of fosfomycin. She was advised to discontinue the use of phenazopyridine and to avoid this medication in the future. She was then discharged home without requiring any supplemental oxygen and with plans to follow up with her primary care provider in three weeks.

Upon return of send-out labs, her methemoglobin levels were found to be 0%, but there was evidence of significantly elevated levels of sulfhemoglobinemia. The patient was asymptomatic at the time of her outpatient follow-up and was advised to avoid sulfur-containing drugs in the future.

## Discussion

**Figure 1:**
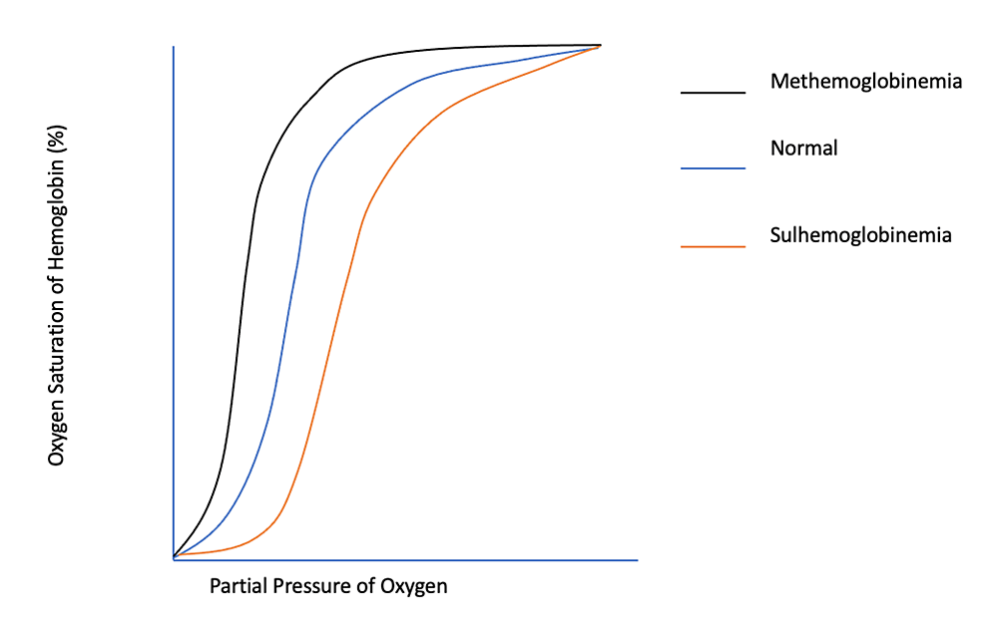
Hemoglobin Dissociation Curve Image Credits: Matthew A. Soderstrom

Dyshemoglobinemia should be suspected when a patient presents with symptoms of cyanosis accompanied by low pulse oximetry readings refractory to the administration of supplemental oxygen. The two most common dyshemoglobinemias are methemoglobinemia and sulfhemoglobinemia. Methemoglobinemia is more frequently diagnosed and is caused by the oxidation of heme iron leading to the ferric iron state (Fe3+), which has a high affinity for oxygen, causing a leftward shift in the oxygen-hemoglobin dissociation curve (Figure 1) [2].

Methemoglobinemia is most often induced via oxidizing medications such as local anesthetics and sulfur-based antibiotics [3,4]. This process can typically be reversed with the reducing agent methylene blue, which allows heme iron to return to its original oxidation state with subsequent resolution of cyanosis via increased oxygen delivery at peripheral tissues [5]. Sulfhemoglobinemia, unlike methemoglobinemia, occurs not by reduction or oxidation of the existing heme molecule but by the insertion of a sulfur atom directly into the heme structure [5]. This atomic rearrangement is irreversible and results in a molecule - sulfhemoglobin - that is incapable of carrying oxygen. However, the sulfur atom binds to only one or two of the subunits of the heme tetramer and reduces the affinity of the remaining heme moieties for oxygen [6]. This allows for the release of oxygen molecules, which can facilitate oxygenation in the peripheral tissues until high concentrations of sulfhemoglobin are present in the blood. This relationship between the sulfur atom and the heme tetramer manifests as a right shift in the oxygen-hemoglobin dissociation curve, in direct contrast to the pathophysiologic mechanism of methemoglobinemia. Therefore, while sulfhemoglobinemia is able to cause extensive cyanosis at relatively low concentrations (0.5mg/dL), it rarely manifests with symptoms of true hypoxia [7]. However, patients may become symptomatic if sulfhemoglobin is present at high enough levels in the blood or in the setting of comorbid anemia.

There are numerous prescription medications and over-the-counter drugs that have been implicated in the development of sulfhemoglobinemia (Table 2). Each of these substances increases the amount of sulfur oxide within the blood, either through direct interaction of the offending molecule with the heme tetramer or indirectly through the increase in endogenous levels of sulfur-producing bacteria [8]. Phenazopyridine is often used as an adjunct to antimicrobial therapy in the treatment of urinary tract infections. While the mechanism is not fully understood, phenazopyridine has been hypothesized to exert an analgesic effect on the urinary mucosa, which lessens the symptoms of urgency, frequency, and dysuria associated with symptomatic urinary tract infections [9]. The drug instructions state that the usual dose for adults is 200 mg three times a day for a maximum of two days from symptom onset. While the mechanism is unclear, there are multiple examples of phenazopyridine-induced sulfhemoglobinemia in the literature.

**Table 2:** Common Agents Implicated in Sulfhemoglobinemia

Drug Class	Drug
Analgesic	Phenazopyridine [10], Phenacetin [1], Acetanilidie [1]
Antianginal	Nitrates [11]
Antimigraine	Sumatriptan [10]
Antiemetic	Metoclopramide [12]
Antimicrobial	Trimethoprim-sulfamethoxazole [13], Dapsone [14]

Confirmation of sulfhemoglobinemia can be challenging. Many hospitals have co-oximeters or arterial blood gas analyzers with built-in spectrometers that can detect deoxyhemoglobin, oxyhemoglobin, carboxyhemoglobin, and methemoglobin [15]. Sulfhemoglobin shares a similar wavelength with methemoglobin, and it is, therefore, difficult to distinguish between these two molecules. In most cases, confirmation of sulfhemoglobinemia ultimately requires a send-out lab, as most institutions do not have in-house capability for sulfhemoglobin detection. Given the relative ease of ruling out methemoglobinemia, awareness of alternative forms of dyshemoglobinemias is important to avoid rejecting these disorders when considering a patient’s differential diagnosis.

## Conclusions

The recognition, diagnosis, and treatment of hypoxia are essential skills for both internists and critical care physicians. While uncommon, dyshemoglobinemias are important etiologies to consider when there is discordance between pulse oximetry and the physical exam, especially when pulse oximetry is not improving with supplemental oxygen. While methemoglobinemia is much more common, sulfhemoglobinemia and the drugs associated with the disease are a diagnosis internists and critical care physicians must be familiar with.
